# δ^15^N Values in *Crassostrea* virginica Shells Provides Early Direct Evidence for Nitrogen Loading to Chesapeake Bay

**DOI:** 10.1038/srep44241

**Published:** 2017-03-10

**Authors:** H. D. Black, C. F. T. Andrus, W. J. Lambert, T. C. Rick, D. P. Gillikin

**Affiliations:** 1 Department of Geological Sciences, University of Alabama, Tuscaloosa, AL 35487, USA; 2 Department of Earth and Environment, Florida International University, Miami, Florida 33199, USA; 3 Department of Anthropology, NHB 112, National Museum of Natural History, Smithsonian Institution, Washington, DC 20013, USA; 4 Department of Geology, Union College, Schenectady, NY 12308, USA.

## Abstract

*Crassostrea virginica* is one of the most common estuarine bivalves in the United States’ east coast and is frequently found in archaeological sites and sub-fossil deposits. Although there have been several sclerochronological studies on stable carbon and oxygen isotopes in the shells of this species, less is known about δ^15^N values within their shells, which could be a useful paleoenvironmental proxy to assess estuarine nitrogen dynamics. Modern *C. virginica* samples were collected in Chesapeake Bay for comparison with archaeological shells from nearby sites ranging in age from ~100 to 3,200 years old. Left valves were sampled by milling the hinge area and the resulting powder was analyzed for %N and δ^15^N values. Comparison of δ^15^N values between *C. virginica* shells shows relatively constant values from ~1250 BC to ~1800 AD. After ~1800 AD, there are rapid increases in ^15^N enrichment in the shells, which continue to increase in value up to the modern shell values. The increase in δ^15^N values is evidence of early anthropogenic impact in Chesapeake Bay. These results corroborate the observation that coastal nitrogen pollution occurred earlier than the 19th century and support the use of oyster shell δ^15^N values as a useful environmental proxy.

Anthropogenic pollution, especially nitrogen, is a major concern in coastal estuaries^1^,^2^,^3^. Increased nitrogen loading can potentially lead to eutrophication, which can cause anoxic conditions in water bodies and die-off events of marine organisms in dead zones^4^,^5^,^6^. Since the 1970s, regulations governing nitrogen inputs have been introduced, but in order for these policies to be effective, it is imperative to know base levels of nitrogen in the area. In most cases, however, base levels before anthropogenic influences began are unknown. If mollusk shells serve as a paleoenvironmental proxy for environmental N levels using stable nitrogen isotope (δ^15^N) values, scientists could generate a base level of nitrogen by studying the record of nitrogen isotopes stored in the shells, as well as how nitrogen sources and concentrations have fluctuated over time.

Bivalve shell geochemistry is commonly used to assess environmental parameters at the time the shell grew, such as sea surface temperature and salinity^7^,^8^,^9^,^10^,^11^,^12^. The eastern oyster *Crassostrea virginica* has large environmental tolerances; able to survive significant variations in temperature (20–45 °C), salinity (0–42 psu), and food supply^13^. Since they are sessile filter feeders in physical contact with water, their soft tissues and shells can record environmental changes by incorporating organic materials from the surrounding water body^14^,^15^. *C. virginica* shells are primarily calcitic, but have a thin aragonite layer in the ligostracum and muscle scar^16^. This species has previously been used for numerous stable isotope studies on stable carbon and oxygen isotopes within the shell (e.g. refs 17, 18, 19, 20), and recently has been introduced as a possible paleoenvironmental proxy for environmental N levels by analyzing the shell δ^15^N values^15^,^21^,^22^,^23^. Anthropogenic nitrogen sources, specifically sewage and industrial pollutants, are typically more ^15^N enriched than non-anthropogenic sources^24^,^25^,^26^, making it possible to trace nitrogen inputs into estuaries by studying the δ^15^N values in the tissue and shell organic fraction.

Unlike the soft tissue samples that were traditionally used in biological and environmental studies of mollusks, shell material can potentially serve as a better proxy for past conditions due to the increased chance of preservation over time. Mollusk shell material accretes sequentially, sometimes in annual growth layers, which could potentially be used as a high resolution temporal proxy for historical nitrogen levels, as well as creating a time-series of nitrogen loading over the life of the organism^11^,^26^,^27^. Furthermore, the shell material is never metabolized, so it can record the environmental conditions over a longer period of time than soft tissues^12^,^26^,^27^. It has previously been established that the calcite shell organic fraction chemistry reflects that of the organisms’ soft tissues, with minimal variability in δ^15^N values (~0–2‰) between the soft tissues and shell material^27^,^28^.

Here we determine if *C. virginica* shells record historical evidence of N levels in the Rhode River basin in Chesapeake Bay during the late Holocene to assess when anthropogenic N loading began. In doing so, we seek to better validate and establish this novel environmental proxy. To test for Chesapeake Bay nitrogen in historic and prehistoric mollusk shells, we sampled archaeological and modern (living) oysters from the Rhode River Estuary at the Smithsonian Environmental Research Center in Edgewater, Maryland (38°53′N 76°32′W) (Fig. 1). Our archaeological samples date to the Woodland Period (including the Early [1200–500 BC], Middle [500 BC-AD 900], and Late [AD 900–1600]), and the 18^th^ and 19^th^ centuries^21^. During the Woodland period in the Chesapeake, Native American peoples practiced a largely hunting and gathering lifestyle and their populations appeared to expand during the Middle Woodland and especially during the Late Woodland, when maize agriculture appeared in parts of the region^29^,^30^,^31^. European colonization intensified in the area in the 17^th^ century, with colonial settlements clearing the forests and establishing large plantations on pristine agricultural land^32^,^33^. During the 19^th^ century, industrialization rapidly increased in the eastern US and continues today^33^.

Although Native Americans practiced agriculture and cleared forests in the region, these impacts were more localized and less widespread than the major landscape clearance efforts of Euro-American colonists and later peoples from the 17^th^ century onward. Historic period land clearance resulted in significant sedimentation^34^, and likely nutrient loading, into the Bay. These data lead Kirby and Miller^34^ to hypothesize that an increase in observed growth rates of oysters from archaeological sites on the Potomac and Patuxent rivers during the 18^th^ and early 19^th^ century may have been due to an increase in planktonic primary productivity from nutrient loading during early eutrophication. The initial period of faster growth was later followed by a size decrease that they speculate was from intensive eutrophication and environmental deterioration, such as increased occurrences of harmful algae and parasitic disease after AD 1860. They speculate that other environmental changes, such as changes in water chemistry and an increase in the degree of fishing disturbances, in Chesapeake Bay during this time could affect the growth rate of *C. virginica*, but these environmental conditions are unlikely to cause as significant of an effect in shell growth rates as eutrophication. Our δ^15^N data provide a means to test this hypothesis and to help develop a novel proxy for assessing N in marine shellfish.

## Results

One-way ANOVA tests for δ^15^N values between time periods produced an F-value of 297.3 and a significance of p < 0.001 at α = 0.05 (data summarized in Table 1). This indicates that the sample variance between different time periods was greater than the variance within time periods. A Tukey Post Hoc test yielded results that show that archaeological samples from the Early Woodland (1250–800 BC) are significantly different from Middle Woodland (500 BC-AD 900), 19^th^ century, and modern samples. Archaeological samples from the Middle Woodland were significantly different from Early Woodland, latter 19^th^ century, and modern samples. Archaeological samples from the Late Woodland (AD 900–1600), 18^th^ and early 19^th^ century were significantly different from latter 19^th^ century and modern samples. Later 19^th^ century and modern shells were significantly different from all other time periods (Fig. 2).

The F-value calculated by a one-way ANOVA for percent nitrogen (%N) was 34.8 with a significance of p < 0.001 at α = 0.05. A Tukey Post Hoc test yielded results that show archaeological samples from the Early, Middle, and Late Woodland periods were significantly different only from the modern samples (data summarized in Table 2). The 18^th^ and 19^th^ century samples were only significantly different from the modern samples and the modern samples were significantly different from all other time periods (Fig. 3).

## Discussion

Our results indicate increased N loading to Chesapeake Bay started as early as the 19^th^ century and substantially increased to the present. Time-series comparisons of the sampled shells display distinct trends, with both %N and δ^15^N values following a roughly exponential curve with higher concentrations of N and more ^15^N enriched shells occurring in the 19^th^ century and modern samples. The %N remains more constant than the δ^15^N values, but increases ~0.07% from the 18^th^ century to modern shells (Fig. 3). The δ^15^N values remain relatively constant from the Early Woodland until the first part of the late 18^th^ to 19^th^ century (i.e., 1750–1800) but then increase ~3‰ from the late 18^th^ century to mid late 19^th^ century (i.e., 1850–1900) (Fig. 2). There was another substantial increase in δ^15^N values (~1‰) between the mid to late 19^th^ century shells and the modern collected shells, which is correlated to a 10-fold increase in reactive N species globally from 1860 to 1990^35^.

δ^15^N values in mollusk shells have been shown to faithfully track the values in soft tissues and ambient POM^26^,^27^,^36^. In contrast, variation in mollusk shell %N has not been validated as a meaningful proxy for ambient N concentrations and additional analysis of modern samples is required before interpretation of such data is possible. Although the relatively constant %N suggests little to no N loss from the shells over time, our discussion will focus solely on δ^15^N values.

We interpret this substantial increase in δ^15^N values to be increased anthropogenic inputs into Chesapeake Bay during the 1800s. During the Woodland Period, it is unlikely that Native Americans^37^ had any significant impact on the N loading of the bay. While there was urban development during the 18^th^ century, the impacts were likely more local in scale, as population levels were comparatively low in much of the region.

Our results are similar to several sediment nutrient loading studies in Chesapeake Bay over the last ~3000 years, with the exception of when δ^15^N values began to rapidly increase within the Bay^38^. Bratton *et al*.^35^ observed significant increases in δ^15^N values from 1750–1800 AD, whereas we determined a later time period of ^15^N enrichment. This is likely due to sampling location, however, since Bratton *et al*.’s study locations were closer to the Susquehanna River, which is a larger source of N for Chesapeake Bay^35^ than our study location. Zimmerman and Canuel^38^ also show increases in δ^15^N values beginning between the 18^th^ and 19^th^ centuries depending on study location within Chesapeake Bay, with their more northerly collected sediment core showing ^15^N enrichment ~100 years before their southern core. A study location farther north in the bay would also not have as long of a period to mix with estuarine waters before deposition, so it could potentially record higher δ^15^N values at an earlier time period. Another possibility for the timing differences between this study and Bratton *et al*.^35^ is due to the dating methods used. While our study solely used ^14^C from corrected and calibrated archaeological oysters^21^, Bratton *et al*.^35^ used pollen stratigraphy, ^14^C of shell material within the cores, and the short-lived radioisotopes ^137^Cs and ^210^Pb within recently deposited sediment^4^. Since pollen was primarily used to relatively date the sediment, it is possible that the absolute dates obtained by their ^14^C analysis could permit larger error in the dating due to reworking and bioturbation of the sediments.

During the 19^th^ century, population sizes and industrialization in the northeast US expanded exponentially^39^, which likely had a profound impact on the ecological health of the Bay. Between 1830 and 1880, over 80% of the forest surrounding Chesapeake Bay had been cleared, which, in combination with plowing for agriculture, greatly increased the sediment accumulation in the bay^4^,^5^,^20^,^35^,^39^,^40^,^41^,^42^. During this time, the population size nearby Chesapeake Bay increased threefold^39^, and the increased amounts of sewage discharge and erosion caused by plowing likely increased the N inputs and ^15^N enrichment in the Bay^24^. The significant decrease in oyster populations at this time also contributed to the failing health of the Bay due to decreased filtration rates of the bay water^39^ and increased sedimentation rates which correlates to previous findings of decreased diatom diversity and increase in the centric:pennate diatiom ratio since the beginning of the 19^th^ century^4^. Therefore, it is likely that the POM and sediment of the bay were more enriched in ^15^N. Consequently, we would expect a substantial shift in δ^15^N values during the 19^th^ century due to increased amounts of sewage and eroded soil entering the bay, which was evident in the results of our shell study.

A similar pattern was present in size changes in archaeological oysters. Archaeological size data from throughout the Chesapeake Bay indicate a significant increase in oyster size during the Historic period (18^th^ to 19^th^ centures) when compared to earlier Woodland times^34^,^37^. Research at sites on the Potomac and Patuxent rivers documents this trend in detail, with oysters first increasing in size during the 18^th^ and early 19^th^ centuries and then decreasing dramatically after AD 1860, perhaps from intensive eutrophication^34^. It has been suggested that the degree of organic matter loading into Chesapeake Bay has significantly increased within the last two centuries^38^,^43^,^44^, however, Cornwell *et al*. did not determine substantial increases in N deposition within the sediment, which could be due to increased algal blooms in the water column^38^,^44^ and metabolic activity of estuarine organisms^43^. Our data support this hypothesis, providing the first direct evidence of N loading in this location of the bay during the 19^th^ century, particularly in the latter half of the 19^th^ century.

The potential effects of diagenetic alteration of %N and δ^15^N values must be considered. Loss of shell organic matter, and consequently N, likely occurs after burial and may be evident in the %N trends noted in our study. We argue, however, that such loss would not explain the observed δ^15^N trends. First, the observed trend in δ^15^N values would require preferential loss of ^15^N, which is contradictory to the kinetics of stable isotopes. Secondly, it is difficult to imagine why diagenetic influence of N isotopes would stop in pre-seventeenth century shells, and even reverse to some extent in the earliest samples. Third, Black^28^ was not able to influence shell δ^15^N values during a series of prehistoric cooking methods studies unless the shell was heated to the point at which it began to physically disintegrate. Finally, the close correspondence between our data and the sediment core-based proxies of anthropogenic N noted above indicates that the shell data trends accurately follow levels of environmental N. We argue that the trends in δ^15^N values noted in this study are primarily the result of changes in the sources contributing to the oysters’ habitat. The source for the somewhat higher δ^15^N values in the Early Woodland samples is not readily apparent.

This study demonstrates the potential utility of shell δ^15^N proxies. The abundance of this and similar species in archaeological sites worldwide suggests that detailed records of anthropogenic N impacts can be created from shell midden deposits. Such baseline data are valuable not only in addressing modern pollution concerns, but also in assessing the extent of ancient human habitation, land-use, agricultural practice, and related activities. This approach may be less expensive than sediment core analysis and therefore permit wide geographic coverage to facilitate regional N reconstructions. Additionally, archaeological contexts may allow reliable age control of samples, which is sometimes difficult in cores with disturbed sediments. Sclerochronological shell sampling also permits seasonal analysis of δ^15^N values in large, fast growing species^27^, which provides even more detailed information. Further research is needed to better assess the validity of shell %N data, the impacts of diagenesis and prehistoric cooking methods, and the possible transport of shells before burial.

For the δ^15^N values in *C. viriginica* shells from Chesapeake Bay, there are significant changes in δ^15^N values of the shells over time. The δ^15^N values remain relatively constant from ~3,200 years ago until 1750–1800, but significantly increase after 1850 to the live-collected shells. While there is a possibility that diagenetic processes may have influenced these data, the sharp increase in δ^15^N values between ~1750–1800 and 1850-1900 appears to be primarily due to the industrial revolution, animal wastes, and soil erosion. The subsequent increases from 1850 to 2013 are due to a significant increase in human population, synthetic fertilizer use, sewage discharge, erosion, and other anthropogenic pollution sources in Chesapeake Bay over the last two centuries. Different N pollutants have varying δ^15^N values (i.e., animal wastes range from ~10–20‰ and synthetic fertilizer ~0‰), which explains the large increase in δ^15^N values between ~1750–1800 and 1850–1900 and the smaller increase from ~1850–1900 to modern samples. Between ~1750–1800 and 1850–1900, the main N pollutants in Chesapeake Bay were isotopically enriched, leading to a significant increase in δ^15^N values from earlier periods. The widespread use of synthetic fertilizers in the 20^th^ century likely caused the reduced increase in δ^15^N values between ~1850–1900 to modern samples since synthetic fertilizer has more depleted δ^15^N values than earlier sources while increasing the %N in the Bay.

## Methods

**Figure 1:**
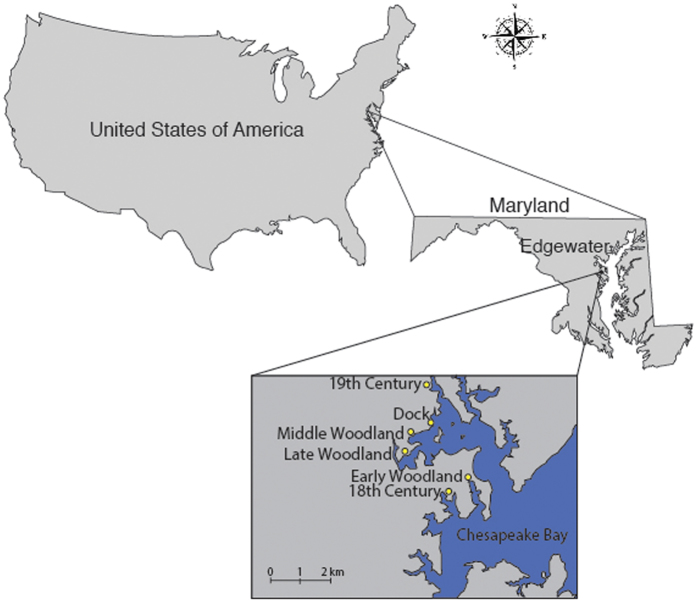
Location of study area near Edgewater, MD, USA. Sample locations are represented by their corresponding archaeological time periods. The site labeled “Dock” denotes the location of modern oyster sampling. Map was created in ArcMap version 10.3 and modified in Adobe Illustrator version CC (http://desktop.arcgis.com/en/arcmap/10.3/main/get-started/whats-new-in-arcgis.htm and http://www.adobe.com/products/illustrator.html, respectively).

**Figure 2:**
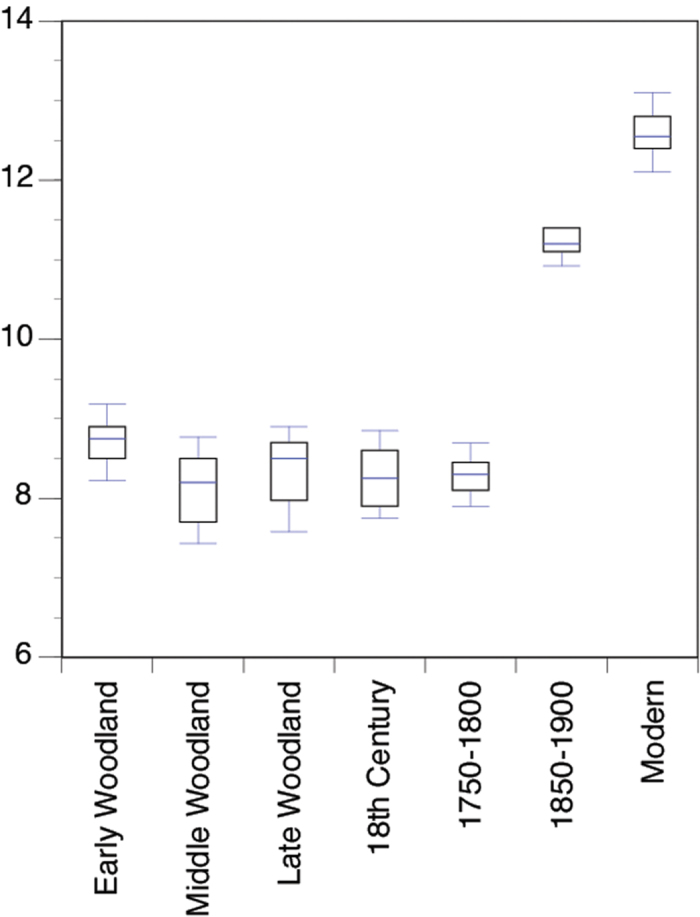
Plot of δ^15^N (‰ AIR) values for modern and archaeological *C. virginica* shells. Precision (1σ) was better than 0.09‰ based on analysis of multiple standards over a range of isotopic values.

**Figure 3:**
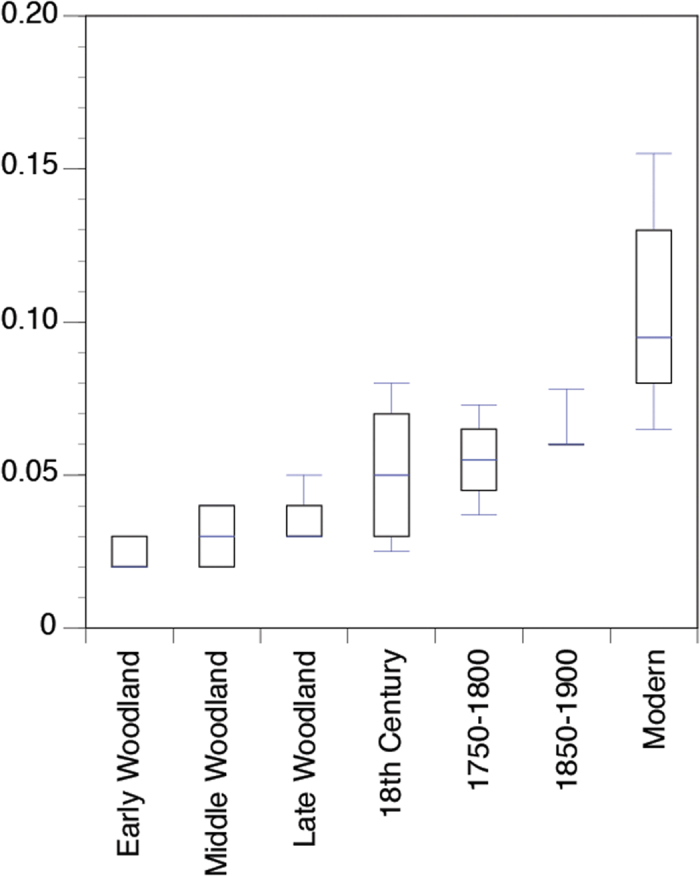
Plot of percent nitrogen values for modern and archaeological *C. virginica* shells.

Oyster shell samples were collected from six different archaeological sites within the Rhode River Estuary of Chesapeake Bay (Fig. 1). Researchers with the Smithsonian Institution collected archaeological samples while excavating shell middens in the region^21^,^37^. Modern oyster samples were collected from an oyster reef located adjacent to one of the archeological sites and in relatively close proximity to the other archaeological sites.

**Figure 4:**
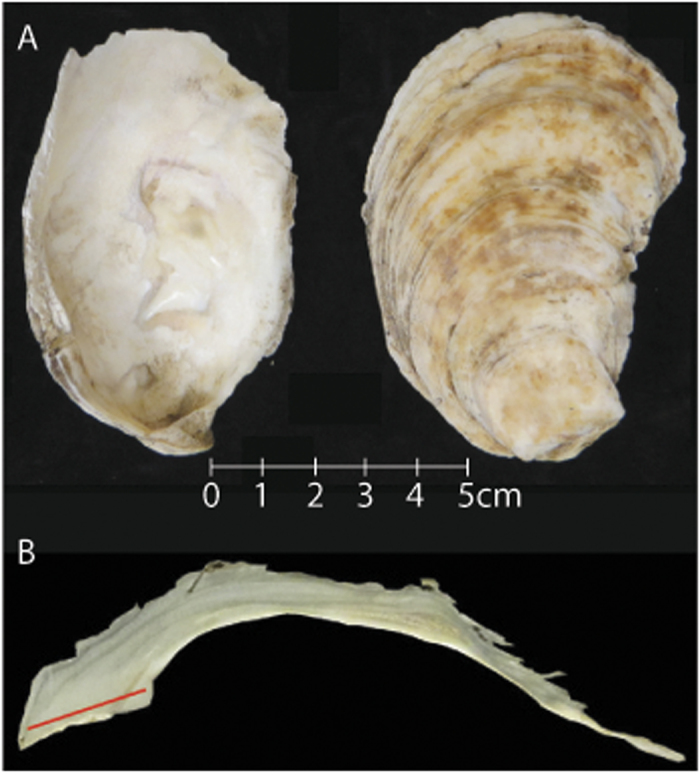
(**A**) Image of disarticulated valves of *C. virginica*. (**B**) Cross section of *C. virginica* through the resilifer. The red line represents the micro-drilled transect.

Ninety archaeological shell samples ranging in age from ~100 to 3,200 years old and thirty modern shell samples were analyzed for %N and δ^15^N values. The ancient shells were all well preserved and robust (i.e., limited taphonomic effects, no noticeable alteration of color of the shell or ligament scar, and non-chalky texture). The soft tissues were removed from the modern shells and the shells were cleaned using DI water and a soft bottlebrush followed by five minutes of ultrasonic cleaning. The shells were dried overnight at room temperature. The left valve of each shell sample was bisected along the longest axis using a slow-speed diamond-wafering saw. Each shell was subsampled using a hand held micro-drill. In order to create a lifetime average of the %N and δ^15^N values, a transect was drilled in cross-section through the resilifer to a depth of approximately 2 mm (Fig. 4). No carbonate powder from the exterior surface of any valve was sampled. Gillikin *et al*.^27^ has shown that the oxidative cleaning required for coral and foraminifera samples is not required for dense bivalve shells. The powdered samples were stored in 4.5 mL round-bottomed borosilicate vials until elemental analyzer –isotope ratio mass spectrometry (EA-IRMS) analysis.

To obtain comparable amounts of nitrogen (i.e., mass spectrometer peak area in millivolts (mV)) between the modern and archaeological shell samples, different masses were used. Between 34–35 mg of powdered samples of modern shells were transferred to 5 × 9 mm tin capsules and 100–110 mg of archeological shells were transferred to 9 × 10 mm tin capsules. The samples were analyzed in the Alabama Stable Isotope Laboratory, in the Department of Geological Sciences at the University of Alabama. Determinations for %N and δ^15^N values were made using a Costech ECS 4010 elemental analyzer (EA) coupled to a Thermo Delta V isotope ratio mass spectrometer without acid pre-treatment of the samples following Versteegh *et al*.^45^. The EA was fitted with a carbon trap [glass trap (Costech part no. 071121) filled with CO_2_ absorbant (Costech part no. 021020)] to remove CO_2_ and operated with a combustion temperature of 1020 °C (reduction column = 650 °C).

The δ^15^N data are reported in parts per mil (‰) vs. AIR. Delta notation is calculated using the equation δ^15^N = [(R_sample_ − R_standard_)/R_stantard_] * 1000. Precision (1σ) was better than 0.09‰ based on analysis of two standards over a range of isotopic values (ammonium sulfate standard IAEA-N-2; δ^15^N = 20.3‰) and Elemental Microanalysis high organic sediment standard B2151; δ^15^N = 4.4‰). Two elemental standards, acetanilide (%N = 10.36%) and B2151 (%N = 0.5%), were used to build the calibration curve for each run and check for accuracy, respectively. Thirty-four samples were analyzed in duplicate with precision better than 0.07‰. One-way ANOVA and Tukey Post Hoc tests were completed on the shell samples based on time period to determine if there were any statistically significant differences between periods over time.

## Additional Information

**How to cite this article:** Black, H. D. *et al*. δ^15^N Values in *Crassostrea* virginica Shells Provides Early Direct Evidence for Nitrogen Loading to Chesapeake Bay. *Sci. Rep.* **7**, 44241; doi: 10.1038/srep44241 (2017).

**Publisher's note:** Springer Nature remains neutral with regard to jurisdictional claims in published maps and institutional affiliations.

## Figures and Tables

**Table 1:** δ^15^N (‰ AIR) values for the periods sampled [Archaeological periods Early Woodland (Site 18AN308), Middle Woodland (Site 18AN285), Late Woodland (Site 18AN287), 18^th^ Century (Site 18AN839), 19^th^ Century (Site 18AN1323), and Modern].

Archaeological Site	Calibrated Age (AD/BC, 2σ)*	Average δ^15^N (‰ AIR)	Minimum δ^15^N (‰ AIR)	Maximum δ^15^N (‰ AIR)	Number of Samples
Early Woodland	1250–840 BC	8.8	8.2	9.9	16
Middle Woodland	AD 550–680	8.2	7.0	8.9	18
Late Woodland	AD 1290–1510	8.3	6.5	8.9	19
18^th^ Century	AD 1710–1850	8.3	7.6	9.0	10
19^th^ Century	AD 1850–1950	11.2	10.9	11.4	19
Modern	AD 2012–2013	12.6	11.8	13.6	30

Precision (1σ) was better than 0.09‰.

^*^All dates calibrated using OxCal 4.2^35^,^36^ and applying a standard reservoir correction of 97 ± 18 years for all shells. Details for all site chronologies are provided in Rick *et al*.^21^.

^1^Ages given in text and figures are based on radiocarbon dates, stratigraphic position, and presence of time sensitive artifacts (see refs 21 and 37).

**Table 2:** Percent nitrogen values for the periods sampled [Archaeological periods Early Woodland (Site 18AN308), Middle Woodland (Site 18AN285), Late Woodland (Site 18AN287), 18^th^ Century (Site 18AN839), 19^th^ Century (Site 18AN1323), and Modern].

Archaeological Site	Calibrated Age (AD/BC, 2σ)*	Average %N	Minimum %N	Maximum %N	Number of Samples
Early Woodland	1250–840 BC	0.05	0.02	0.09	16
Middle Woodland	AD 550–680	0.06	0.03	0.08	18
Late Woodland	AD 1290–1510	0.02	0.01	0.05	19
18^th^ Century	AD 1710–1850	0.04	0.02	0.05	10
19^th^ Century	AD 1850–1950	0.03	0.02	0.05	19
Modern	AD 2012–2013	0.11	0.05	0.23	30

^1^Ages given in text and figures are based on radiocarbon dates, stratigraphic position, and presence of time sensitive artifacts (see refs 21 and 37).

## References

[b1] Fisher D. & Oppenheimer M. Atmospheric nitrogen deposition and the Chesapeake Bay estuary. Ambio. 20, 102–108 (1991).

[b2] McClelland J. W. & Valiela I. Linking nitrogen in estuarine producers to land-derived sources. Limnology and Oceanography. 43, 577–585 (1998).

[b3] McKinney R. A., Lake J. L., Charpentier M. A. & Ryba S. A. Using mussel isotope ratios to assess anthropogenic nitrogen inputs to freshwater ecosystems. Environmental Monitoring and Assessment. 74, 167–192 (2002). 11878641 10.1023/a:1013824220299

[b4] Cooper S. R. & Brush G. S. A 2,500-year history of anoxia and eutrophication in Chesapeake Bay. Estuaries. 16, 617–626 (1993).

[b5] Howarth R. W. Coastal nitrogen pollution: A review of sources and trends globally and regionally. Harmful Algae. 8, 14–20 (2008).

[b6] Watanabe S., Kodama M. & Fukuda M. Nitrogen stable isotope ratio in the Manila clam, *Ruditapes phillippinarum*, reflects eutrophication levels in tidal flats. Marine Pollution Bulletin. 58, 1447–1453 (2009). 19647270 10.1016/j.marpolbul.2009.06.018

[b7] Epstein S., Bucksbaum R., Lowenstam H. A. & Urey H. C. Revised carbonate-water isotopic temperature scale. Geological Society of America Bulletin. 64, 1315–1326 (1953).

[b8] Emiliani C., Cardini L., Mayeda T., McBurney C. B. M. & Tongiorgi E. Paleotemperature analysis of marine molluscs (food refuse) from the site of Arene Candide Cave, Italy and the Haua Fteah Cave, Cyrenaica. In: Isotopic and cosmic chemistry(ed. Craig H., Miller S. L. & Wasserburg G. J.). (North Holland, Amsterdam, 1964).

[b9] Grossman E. L. & Ku T. I. Oxygen and carbon isotope fractionation in biogenic aragonite: Temperature effects. Chemical Geology. 59, 59–74 (1986).

[b10] Bailey G. N., Deith M. R. & Shackleton N. J. Oxygen isotope analysis and seasonality determinations: Limits and potential of a new technique. American Antiquity. 48, 390–398 (1983).

[b11] Jones D., Quitmyer I., Arnold W. & Marelli D. Annual shell banding, age, and growth rate of hard clams (*Mercenaria spp.*) from Florida. Journal of Shellfish Research. 9, 215–225 (1990).

[b12] O’Donnell T. H., Macko S. A., Chou J., Davis-Hartten K. L. & Wehmiller J. F. Analysis of δ^13^C, δ^15^N, and δ^34^S in organic matter from the biominerals of modern and fossil *Mercenaria spp*. Organic Geochemistry. 34, 165–183 (2003).

[b13] Galtsoff P. S. The American oyster: *Crassostrea virginica* Gremlin. Fishery Bulletin of the Fish Wildlife Service. 64, 1–480 (1964).

[b14] Dame R. F., Spurrier J. D. & Wolaver T. G. Carbon, nitrogen, and phosphorus processing by an oyster reef. Marine Ecology Progress Series. 54, 249–256 (1989).

[b15] Carmichael R. H., Annett B. & Valiela I. Nitrogen loading to Pleasant Bay, Cape Cod: Application of models and stable isotopes to detect incipient nutrient enrichment of estuaries. Marine Pollution Bulletin. 48, 137–143 (2004). 14725885 10.1016/S0025-326X(03)00372-2

[b16] Andrus C. F. T. & Crowe D. E. Geochemical analysis of *Crassostrea virginica* as a method to determine season of capture. Journal of Archaeological Science. 27, 33–42 (2000).

[b17] Andrus C. F. T. Isotope sclerochronology in southeastern US archaeology to estimate season of capture. In: Seasonality and human mobility along the Georgia Bight (ed. Reitz E. J., Quitmyer I. R. & Thomas D. H.) American Museum of Natural History Anthropological Papers(2012).

[b18] Harding J. M., Spero H. J., Mann R., Herbert G. S. & Silko J. L. Reconstructing early 17th century estuarine drought conditions from Jamestown oysters. Proceedings of the National Academy of Sciences. 107, 10549–10554 (2010). 10.1073/pnas.1001052107 PMC2890847 20534581

[b19] Thompson V. & Andrus C. F. T. Evaluating mobility, monumentality, and feasting at the Sapelo Island shell ring complex. American Antiquity. 76, 315–344 (2011).

[b20] Bronk Ramsey C. Bayesian analysis of radiocarbon dates. Radiocarbon. 51, 337–360 (2009).

[b21] Rick T. C. . Shell middens, cultural chronologies, and coastal settlement on the Rhode River sub-estuary of Chesapeake Bay, Maryland, USA. Geoarchaeology. 29, 371–388 (2014).

[b22] Surge D., Lohmann K. C. & Dettman D. L. Controls on the isotopic chemistry of the American oyster, *Crassostrea virginica*: Implications for growth patterns. Palaeogeography, Palaeoclimatology, Palaeoecology. 172, 283–296 (2001).

[b23] Darrow E. S., Carmichael R. H., Andrus C. F. T. & Jackson H. E. From middens to modern estuaries, oyster shells sequester source-specific nitrogen. Geochimica Et Cosmochimica Acta. 202, 39–56 (2016).

[b24] Heaton T. H. E. Isotopic studies of nitrogen pollution in the hydrosphere and atmosphere: A review. Chemical Geology. 59, 87–102 (1986).

[b25] Fritts A. K., Fritts M. W., Haag W. R., DeBoer J. A. & Casper A. F. Freshwater mussel shells (Unionidae) chronicle changes in a North American river over the past 1000 years. Science of the Total Environment. 575, 199–206 (2017). 27741455 10.1016/j.scitotenv.2016.09.225

[b26] Carmichael R. H., Hattenrath T., Valiela I. & Michener R. H. Nitrogen stable isotopes in the shell of *Mercenaria mercenaria* trace wastewater inputs from watersheds to estuarine ecosystems. Aquatic Biology. 4, 99–111 (2008).

[b27] Gillikin D. P. . High-resolution nitrogen stable isotope schlerochronology of bivalve shell carbonate-bound organics. Geochimica et Cosmochimica Acta. 200, 55–66 (2017).

[b28] Black H. B. δ^15^N in mollusk shells as a potential paleoenvironmental proxy for nitrogen loading in Chesapeake Bay. (University of Alabama, 2014).

[b29] Dent R. J. Chesapeake prehistory: Old traditions, new directions. (Plenum, New York, 1995).

[b30] Potter S. R. Commoners, tribute, and chiefs: The development of the Algonquian culture in the Potomac Valley. (University Press of Virginia, 1993).

[b31] Gallivan M. The archaeology of native societies in the Chesapeake: New investigations and interpretations. Journal of Archaeological Research. 19, 281–325 (2011).

[b32] Miller H. M. Living along the “Great Shellfish Bay”: The relationship between prehistoric peoples and the Chesapeake. In Discovering the Chesapeake: The history of an ecosystem(ed. Curtin D., Brush G. S. & Fisher G.). (Johns Hopkins University Press, 2001).

[b33] Miller H. M. Transforming a “splendid and delightsome land”: Colonists and ecological change in the Chesapeake 1607-1820. Journal of the Washington Academy of Sciences. 76, 173–187 (1986).

[b34] Kirby M. X. & Miller H. M. Response of a benthic suspension feeder (*Crassostrea virginica* gmelin) to three estuaries of anthropogenic eutrophication in Chesapeake Bay. Estuarine, Coastal, and Shelf Science. 62, 679–689 (2005).

[b35] Bratton J. F., Colman S. M. & Seal R. R. Eutrophication and carbon sources in Chesapeake Bay over the last 2700 yr: Human impacts in context. Geochimica Et Cosmochimica Acta. 67, 3385–4402 (2003).

[b36] Fertig B., Carruthers T. J. B., Dennison W. C., Fertig E. J. & Altabet M. A. Eastern oyster (*Crassostrea virginica*) δ^15^N as a bioindicator of nitrogen sources: Observations and modeling. Marine Pollution Bulletin. 60, 1288–1298 (2010). 20381097 10.1016/j.marpolbul.2010.03.013 PMC3619001

[b37] Rick T. C. . Millennial-scale sustainability of the Chesapeake Bay Native American oyster fishery. Proceedings of the National Academy of Sciences. 113, 6568–6573 (2016). 10.1073/pnas.1600019113 PMC4988611 27217572

[b38] Zimmerman A. R. & Canuel E. A. Sediment geochemical records of eutrophication in the mesohaline Chesapeake Bay. Limnology and Oceanography. 47, 1084–1093 (2002).

[b39] Kemp W. M. . Eutrophication of Chesapeake Bay: Historical trends and ecological interactions. Marine Ecology Progress Series. 303, 1–29 (2005).

[b40] Reimer P. J. . IntCal09 and Marine09 radiocarbon age calibration curves, 0–50,000 years cal BP. Radiocarbon. 51, 1111–1150 (2009).

[b41] Galloway J. N. . Nitrogen cycles: Past, present, and future. Biogeochemistry. 70, 153–226 (2004).

[b42] Cronin T. . Climatic variability in the eastern United States over the past millennium from Chesapeake Bay sediments. Geology. 28, 3–6 (2000).

[b43] Cornwell J. C., Conley D. J., Owens M. & Stevenson J. C. A sediment chronology of the eutrophication of Cheasapeake Bay. Estuaries. 19, 488–499 (1996).

[b44] Zimmerman A. R. & Canuel E. A. A geochemical record of eutrophication and anoxia in Chesapeake Bay sediments: Anthropogenic influence on organic matter composition. Marine Chemistry. 69, 117–137 (2000).

[b45] Versteech E. A. A., Gillikin D. P. & Dehairs F. Analysis of δ^15^N values in mollusk shell organic matrix by elemental analysis/isotope ratio mass spectrometry without acidification: An evaluation and effects of long-tern preservation. Communications in Mass Spectrometry. 25, 675–680 (2011). 10.1002/rcm.4905 21290456

